# Supplementary material for: WEPP: Phylogenetic placement achieves near-haplotype resolution in wastewater-based epidemiology
Source: PLoS Comput Biol. 2026 Mar 30;22(3):e1014124. doi: 10.1371/journal.pcbi.1014124 (PMC13048486; doi:10.1371/journal.pcbi.1014124)
Supplement: S1 Text — Supplementary Results 1: Incorporating deletions via PanMAT in WEPP has a negligible impact on SARS-CoV-2 variant detection accuracy. Supplementary Results 2: WEPP generalizes to noisy long-read sequencing dataSupplementary Results 3: WEPP accurately distinguishes between recombinants and their parental genomes. In addition, the file includes the following figures: Fig A: Comparison of lineage abundance between WEPP and Freyja on ONT sequenced data.Fig B: Single-nucleotide substitutions separating two haplotypes in a lineage. Fig C: B.1.1.529 (Omicron) Haplotypes detected by WEPP from Point Loma (San Diego) wastewater samples dated December 1, 2, and 5, 2021. Fig D Impact of incorporating deletions in WEPP. Fig E Comparison of the earliest wastewater detection of Omicron haplotypes during the first three weeks of December 2021 with their corresponding earliest clinical collection dates in San Diego. Clinical confirmation was established when the corresponding clinical haplotype was within 1 single-nucleotide substitution of the WEPP-identified haplotype. Fig F Weighted Haplotype Distance and Weighted Peak Distance as a function of (A) sequencing depth, and (B) Phred quality score. Fig G: WEPP’s runtime as a function of sequencing read count and the number of sequences in the MAT. (DOCX) [file pcbi.1014124.s015.docx]

# **Supplementary Material**

**WEPP: Phylogenetic Placement Achieves Near-Haplotype Resolution in Wastewater-Based Epidemiology**

**Authors:**

Pranav Gangwar^1^, Pratik Katte^2^, Manu Bhat^1^, Yatish Turakhia^1,*^

^1^Department of Electrical and Computer Engineering, University of California, San Diego, San Diego, California, USA

^2^Department of Biomolecular Engineering, University of California, Santa Cruz, Santa Cruz, California, USA

^*^ [yturakhia@ucsd.edu](mailto:yturakhia@ucsd.edu)

**Supplementary Results 1: Incorporating deletions via PanMAT in WEPP has a negligible impact on SARS-CoV-2 variant detection accuracy**

Some recently proposed WBE tools, such as Lineagespot[^1^](https://www.zotero.org/google-docs/?iduOBe) and QuaID[^2^](https://www.zotero.org/google-docs/?BA5usn), incorporate deletions in addition to substitutions to detect variants from wastewater. The authors of QuaID claimed that the reduced sensitivity of Freyja, which only considers substitutions in detecting emerging variants of concern, could be due to its lack of consideration of deletions. However, to our knowledge, no study has systematically quantified the impact of deletion inclusion alongside substitutions in SARS-CoV-2 variant detection from wastewater.

We evaluated the impact of considering deletions and substitutions in WEPP by using PanMAT[^3^](https://www.zotero.org/google-docs/?zPI045), a generalized form of MATs that can represent insertions, deletions, and complex mutations in addition to substitutions. To perform this analysis, we constructed a PanMAT that included deletions and substitutions from nearly eight million public SARS-CoV-2 sequences available up to December 25, 2023 (Methods). We excluded insertions for this analysis as it would have required us to define a non-reference-based coordinate system, which would make the analysis quite complex. We then compared WEPP-PanMAT (modified WEPP to support PanMATs), which includes both substitutions and deletions, against WEPP-MAT (default WEPP that uses MATs), which only considers substitutions, on SWAMPy simulated datasets and assessed their accuracy and resolution in variant detection.

The results, shown in Fig D(ii), indicate that incorporating deletions through PanMAT did not improve any of the performance metrics on the SWAMPy simulated data. We attribute this to the relative rarity of deletions compared to substitutions in SARS-CoV-2 sequences, as illustrated in Fig D(i). On average, we find that there are nearly 14 times more substitutions than informative deletions between adjacent lineages (parent and child). These results suggest that deletions contribute minimal additional signal for variant detection, as the numerous substitutions present in the MATs already capture the key distinguishing genomic differences between lineages, and according to Walia et al.[^3^](https://www.zotero.org/google-docs/?TdwlMF), the insertions tend to be even fewer. In other words, our preliminary findings suggest that incorporating insertions and deletions into the WEPP analysis is unlikely to provide major gains.

**Supplementary Results 2: WEPP generalizes to noisy long-read sequencing data**

Wastewater sequencing protocols vary widely across regions and institutions, particularly in terms of library preparation methods and sequencing platforms. This variability makes it essential for any WBE tool to be broadly generalizable and effective across different settings. To evaluate WEPP’s robustness, we tested its performance on Oxford Nanopore Technologies (ONT) long-read sequencing data, which are known to have higher error rates than short-read platforms.

We analyzed three synthetic control mixtures from Ferdous et al.[^4^](https://www.zotero.org/google-docs/?ifALEx), each containing unique combinations of eight known haplotypes spiked into SARS-CoV-2 negative wastewater RNA backgrounds at varying proportions. As shown in Fig A(i) (S1 Table), WEPP achieved a 3.2-fold lower lineage abundance RMSE compared to Freyja, demonstrating strong performance despite the higher noise associated with ONT sequencing.

**Supplementary Results 3: WEPP accurately distinguishes between recombinants and their parental genomes**

Detecting recombinant lineages in wastewater samples is epidemiologically important, as it enables public health officials to monitor pathogen evolution in near real time and provides early warning for emerging variants. We therefore evaluated WEPP’s ability to detect recombinants in wastewater under two scenarios: (1) when the recombinant lineage is *known*, i.e., present in the MAT, and (2) when the recombinant is *novel*, i.e., not present in the MAT.

Wastewater mixtures were simulated using ART, with the recombinant haplotype and its two parental lineage haplotypes in equal proportions (33% each). We simulated two recombinant mixtures: one involving a Delta–Omicron recombinant (XAY) and another involving an Omicron–Omicron recombinant (XBF).

The results, summarized in S12 Table, show that when the recombinant lineage is *known*, WEPP accurately recovers the proportions of all lineages and their corresponding haplotypes. The selected haplotypes are within a single-nucleotide substitution from the simulated haplotypes. For the case of *novel* recombinant, WEPP still accurately identifies the recombinant’s two parental haplotypes as candidates, and flags as unaccounted alleles nearly all informative sites corresponding to the recombinant haplotype. Analysis of the parsimonious haplotype candidates for these unaccounted alleles shows that most of them included the haplotype closest to the *novel* recombinant as one of the candidates (S12 Table). Only 11 of 75 unaccounted alleles were assigned to a unique haplotype, while the remainder had multiple candidates due to the limited number of informative sites in short sequencing reads.

**Supplementary Figures**

**
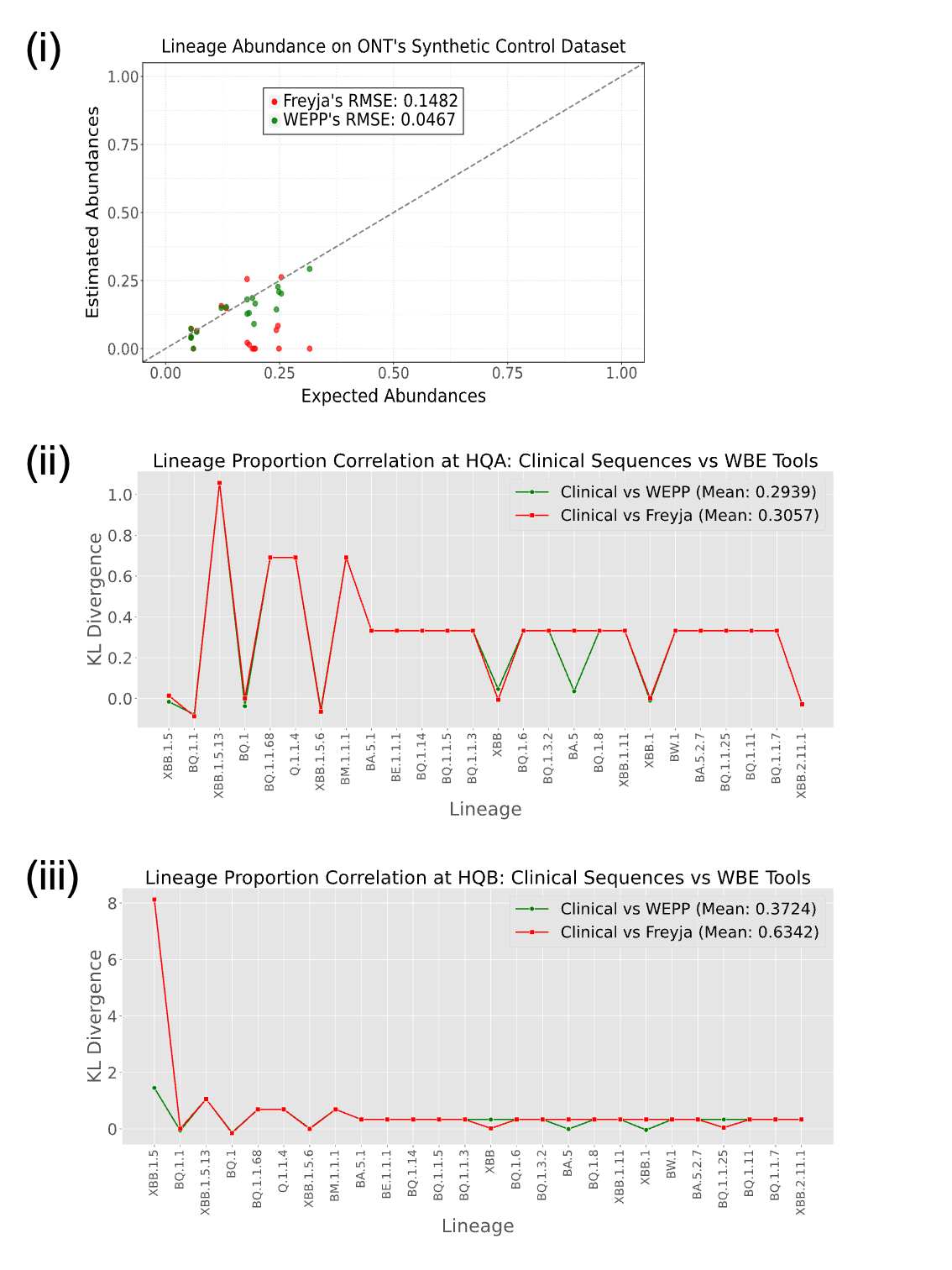
**

**Fig A: Comparison of lineage abundance between WEPP and Freyja on ONT sequenced data.** (i) WEPP and Freyja’s lineage abundance comparison on the ONT sequenced synthetic control dataset. (ii) Average lineage abundance estimates from wastewater samples collected in December 2022 from Hospital Quadrant A, compared with clinical sequences of hospitalized patients in the same hospital and time period[^5^](https://www.zotero.org/google-docs/?1lCKQE). (iii) Average lineage abundance estimates from wastewater samples collected in December 2022 from Hospital Quadrant B, compared with clinical sequences of hospitalized patients in the same hospital and time period.


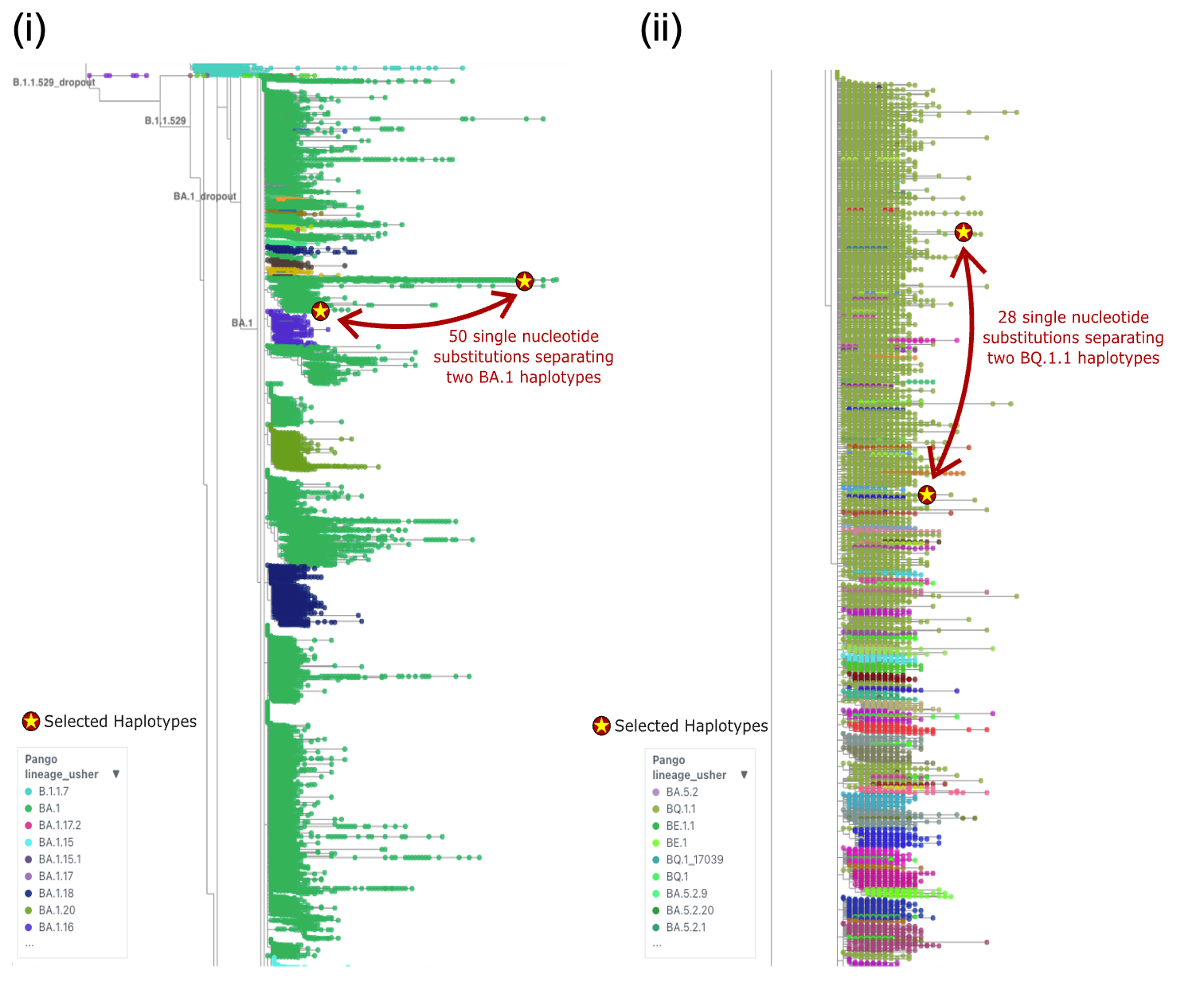


**Fig B: Single-nucleotide substitutions separating two haplotypes in a lineage.** (i) Haplotypes “England/PHEC-5U048Z3E/2022|OX781526.1|2022-01-29” and “England/MILK-3777F8F/2022|OW112938.1|2022-02-18” belonging to lineage BA.1, (ii) Haplotypes “Germany/IMS-10116-CVDP-91FBB221-5948-4641-8459-88ABFE0F8DE1/2023|OY271077.1|2023-03-06” and “USA/PA-CDC-QDX46010440/2023|OQ419762.1|2023-01-24” belonging to BQ.1.1 (right panel)

**Fig C: B.1.1.529 (Omicron) Haplotypes detected by WEPP from Point Loma (San Diego) wastewater samples dated December 1, 2, and 5, 2021.**

**
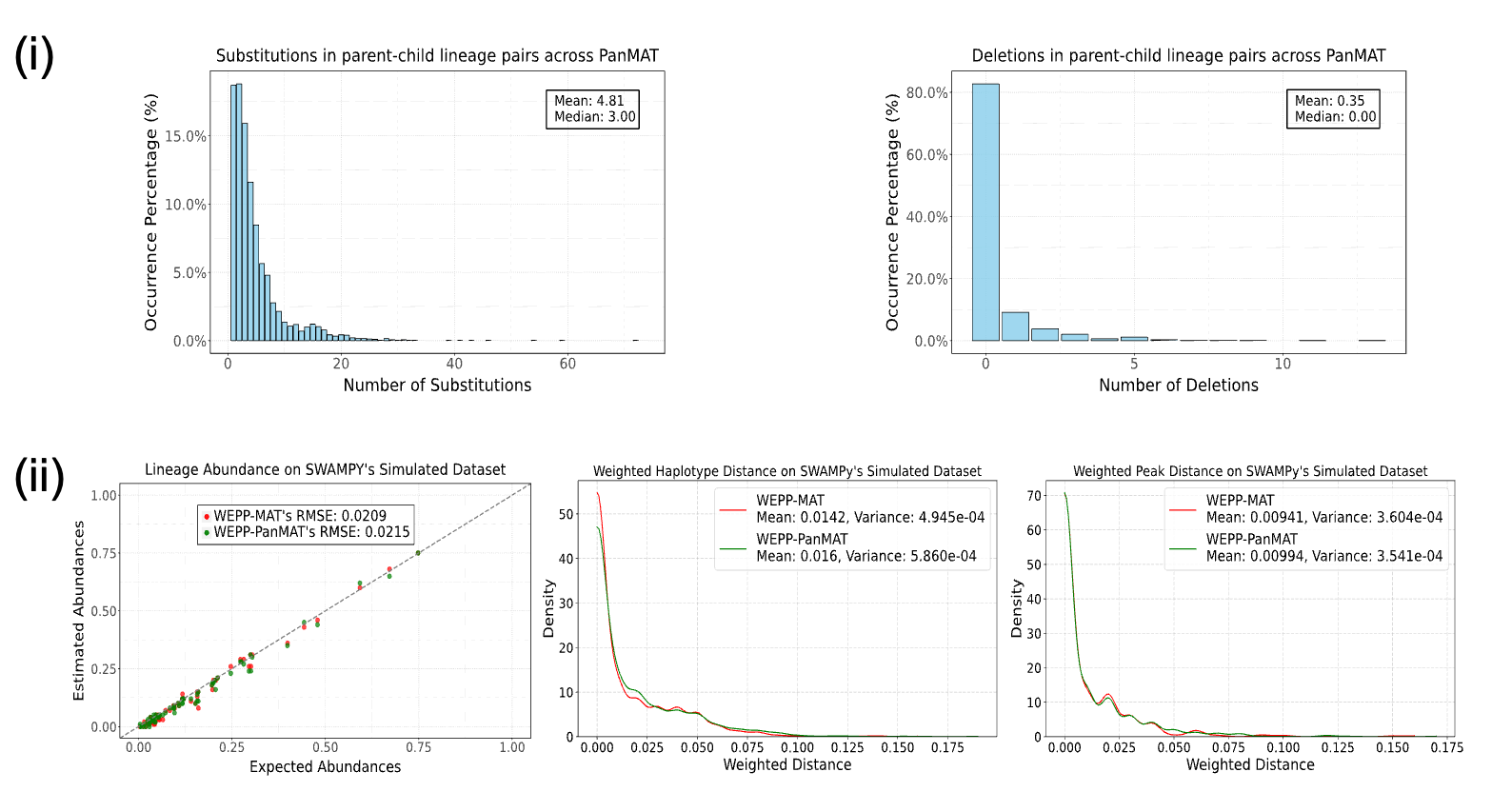
**

**Fig D: Impact of incorporating deletions in WEPP.** (i) Substitution and deletion differences between parent-child lineage pairs in a PanMAT constructed with eight million public sequences available until December 25, 2023. (ii) Performance comparison of WEPP using MAT (WEPP-MAT), which considers only substitutions, and WEPP using PanMAT (WEPP-PanMAT), which includes both substitutions and deletions, based on lineage abundance RMSE (left), Weighted Haplotype Distance (middle), and Weighted Peak Distance (right) on SWAMPy simulated datasets.


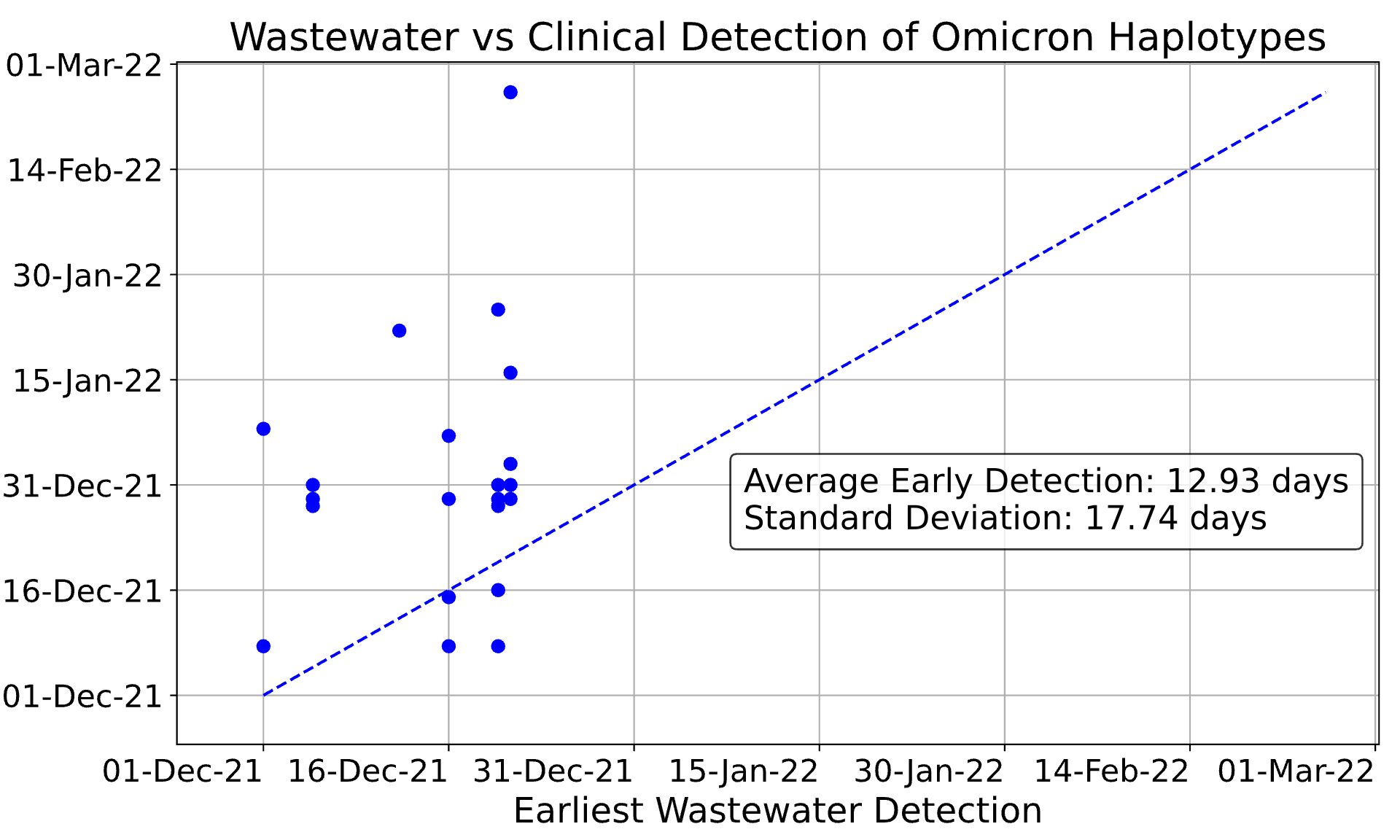


**Fig E: Comparison of the earliest wastewater detection of Omicron haplotypes during the first three weeks of December 2021 with their corresponding earliest clinical collection dates in San Diego. Clinical confirmation was established when the corresponding clinical haplotype was within 0 or 1 single-nucleotide substitution of the WEPP-identified haplotype.**


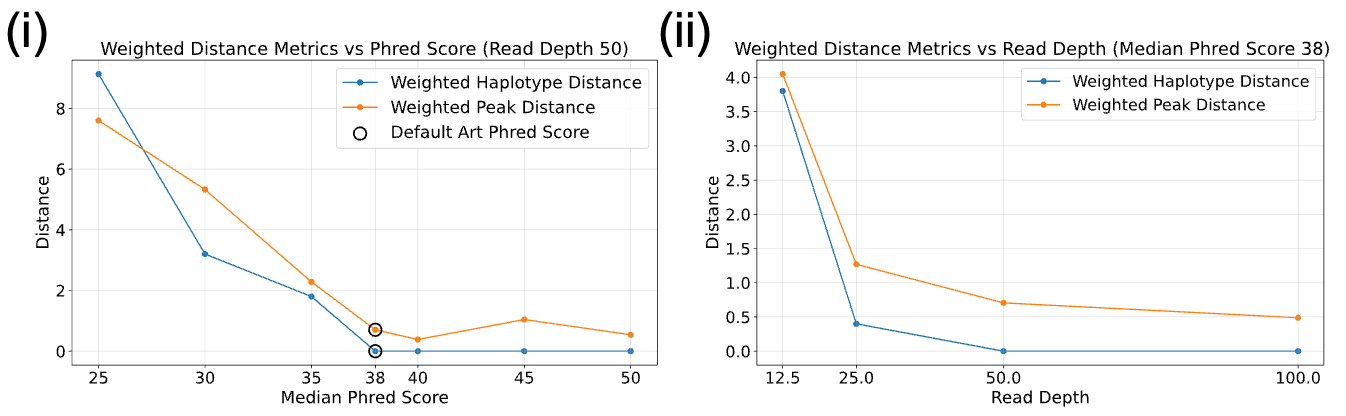


**Fig F: Weighted Haplotype Distance and Weighted Peak Distance as a function of (i) median phred score, and (ii) sequencing read depth.** The wastewater mixtures were simulated using SWAMPy and contained five haplotypes in equal proportions, corresponding to lineages EG.5.1, BQ.1.1.3, JN.1.1, XBB.1.16.15, and XBB.1.5.4. Median phred score was calculated for all based present in the sample and sequencing read depth was calculated by only considering those bases that were above phred score 20 (default masking quality for WEPP). By default, the median phred score of reads in a sample was 38.


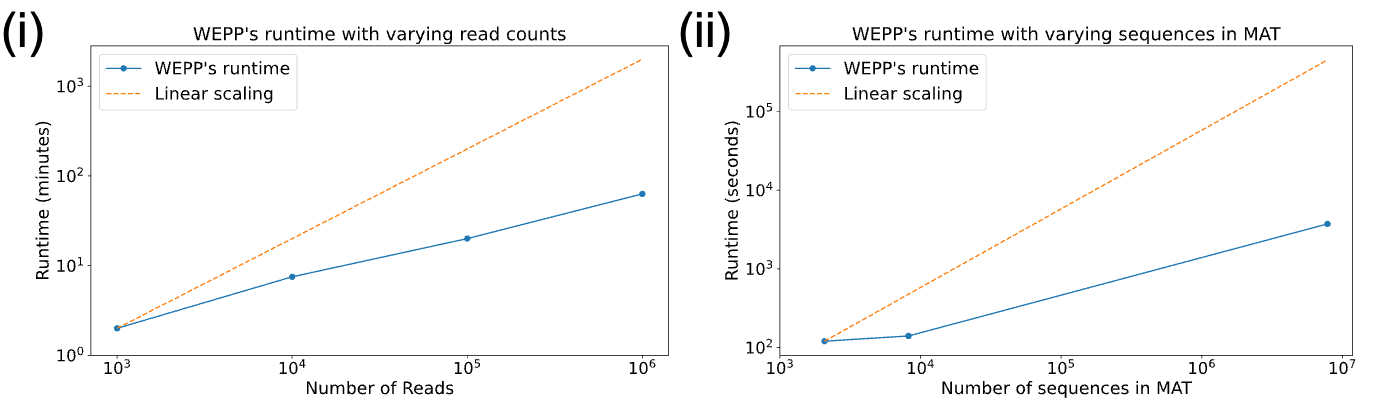


**Fig G: WEPP’s runtime as a function of sequencing read count and the number of sequences in the MAT.** (i) Runtime measured using a SARS-CoV-2 MAT containing 7,840,184 sequences while varying the number of reads from 1k to 1M. (ii) Runtime measured for 1M sequencing reads from human metapneumovirus, respiratory syncytial virus A, and SARS-CoV-2, with MATs containing 2,074, 8,215, and 7,840,184 sequences, respectively.

# **References**

[1. Pechlivanis, N. *et al.* Detecting SARS-CoV-2 lineages and mutational load in municipal wastewater and a use-case in the metropolitan area of Thessaloniki, Greece. *Sci Rep* **12**, 2659 (2022).](https://www.zotero.org/google-docs/?PxCy5B)

[2. Sapoval, N. *et al.* Enabling accurate and early detection of recently emerged SARS-CoV-2 variants of concern in wastewater. *Nat Commun* **14**, 2834 (2023).](https://www.zotero.org/google-docs/?PxCy5B)

[3. Walia, S., Motwani, H., Smith, K., Corbett-Detig, R. & Turakhia, Y. Compressive Pangenomics Using Mutation-Annotated Networks. Preprint at https://doi.org/10.1101/2024.07.02.601807 (2024).](https://www.zotero.org/google-docs/?PxCy5B)

[4. Ferdous, J. *et al.* A gold standard dataset and evaluation of methods for lineage abundance estimation from wastewater. *Science of The Total Environment* **948**, 174515 (2024).](https://www.zotero.org/google-docs/?PxCy5B)

[5. Annavajhala, M. K. *et al.* Hospital wastewater surveillance for SARS-CoV-2 identifies intra-hospital dynamics of viral transmission and evolution. Preprint at https://doi.org/10.1101/2025.03.04.25323323 (2025).](https://www.zotero.org/google-docs/?PxCy5B)
