# Supplementary material for: WEPP: Phylogenetic placement achieves near-haplotype resolution in wastewater-based epidemiology
Source: PLoS Comput Biol. 2026 Mar 30;22(3):e1014124. doi: 10.1371/journal.pcbi.1014124 (PMC13048486; doi:10.1371/journal.pcbi.1014124)
Supplement: Attachment — Submitted filename: Response to Reviewers - WEPP.pdf [file pcbi.1014124.s017.pdf]

We thank the reviewers for their helpful comments and the editor for providing us with the opportunity to submit a revision. We believe that we have addressed the comments thoroughly, which has significantly enhanced our manuscript. Below, we provide a summary of the major changes.

1. **We have added supplementary results comparing the earliest wastewater detection of Omicron haplotypes with their earliest clinical collection dates in San Diego, demonstrating WEPP's early haplotype detection capabilities (Supplementary Fig. 5).**
2. **We have included supplementary results assessing WEPP's accuracy in detecting haplotypes across varying sequencing depths and read quality (Supplementary Fig. 6).**
3. **We have added supplementary results comparing lineage detection capabilities of different tools on simulated and synthetic datasets (Supplementary Table 11).**
4. **We have included results evaluating WEPP's performance in the presence of recombinant lineages (Supplementary Results 3) and added the corresponding results in Supplementary Table 12. This section shows that when the recombinant lineage is *known*, i.e., present in the MAT, WEPP accurately recovers the proportions of all lineages and their corresponding haplotypes. We have also evaluated the case in which the recombinant is *novel*, *i.e.*, not present in the MAT. Here, WEPP selects the recombinant's two parental lineage haplotypes as candidates, and flags as unaccounted alleles nearly all informative sites corresponding to the recombinant haplotype.**
5. **We have added supplementary results measuring WEPP's runtime as a function of sequencing read count and the number of sequences in the MAT (Supplementary Fig. 7).**
6. **We have added supplementary information describing the wastewater sample characteristics used to evaluate WEPP's performance on novel haplotypes, along with a comparison of WEPP's lineage abundance estimates with the ground truth (Supplementary Table 13).**
7. **We have added supplementary information summarizing sample-wise results from Point Loma wastewater data in San Diego, which includes lineages with intra-lineage haplotype clusters, as well as unaccounted alleles absent from clinical sequences from San Diego (Supplementary Table 14).**
8. **We have included a table in the introduction that summarizes the glossary of terms that are used throughout the paper.**
9. **We have included a supplementary information section describing all the supplementary tables.**
10. **We have modified the text to reduce repetitiveness and lengthiness, based on the suggestions of Reviewer-3.**
11. **We have also made minor changes and added clarifications to the text based on the feedback. Changes in the text corresponding to Editor, Reviewer-1, Reviewer-2, and Reviewer-3 are highlighted in red, green, blue, and pink, respectively.**

Below, we provide detailed responses to specific comments.

Editor's Comments

1. Please ensure that the CRediT author contributions listed for every co-author are completed accurately and in full. At this stage, the following Authors/Authors require contributions: Pranav Gangwar, Pratik Katte, Manu Bhatt, and Yatish Turakhia. Please ensure that the full contributions of each author are acknowledged in the "Add/Edit/Remove Authors" section of our submission form. The list of CRediT author contributions may be found here: https://journals.plos.org/ploscompbiol/s/authorship#loc-author-contributions

   We have now completed this information in the online form.

2. We ask that a manuscript source file is provided at Revision. Please upload your manuscript file as a .doc, .docx, .rtf or .tex. If you are providing a .tex file, please upload it under the item type u2018LaTeX Source Fileu2019 and leave your .pdf version as the item type u2018Manuscriptu2019.

   We have uploaded the final manuscript as a .docx file, along with its PDF version and a marked-up PDF copy.

3. Please upload all main figures as separate Figure files in .tif or .eps format. For more information about how to convert and format your figure files please see our guidelines: https://journals.plos.org/ploscompbiol/s/figures

   We have checked our figures with the NAAS tool and have uploaded them separately in the folder 'WEPP figures TIFF'.

4. We have noticed that you have uploaded Supporting Information files, but you have not included a list of legends. Please add a full list of legends for your Supporting Information files after the references list.

   Thanks for letting us know. We have now added a full list of legends for the Supporting Information files after the references list in the main paper.

5. Some material included in your submission may be copyrighted. According to PLOSu2019s copyright policy, authors who use figures or other material (e.g., graphics, clipart, maps) from another author or copyright holder must demonstrate or obtain permission to publish this material under the Creative Commons Attribution 4.0 International (CC BY 4.0) License used by PLOS journals. Please closely review the details of PLOSu2019s copyright requirements here: PLOS Licenses and Copyright. If you need to request permissions from a copyright holder, you may use PLOS's

Copyright Content Permission form.
Please respond directly to this email and provide any known details concerning your material's license terms and permissions required for reuse, even if you have not yet obtained copyright permissions or are unsure of your material's copyright compatibility. Once you have responded and addressed all other outstanding technical requirements, you may resubmit your manuscript within Editorial Manager.

Potential Copyright Issues:
i) Figure 1. Please confirm whether you drew the images / clip-art within the figure panels by hand. If you did not draw the images, please provide (a) a link to the source of the images or icons and their license / terms of use; or (b) written permission from the copyright holder to publish the images or icons under our CC BY 4.0 license. Alternatively, you may replace the images with open source alternatives. See these open source resources you may use to replace images / clip-art:
- https://commons.wikimedia.org
- https://openclipart.org/.

ii) The following Figure contains screenshots: 1. We are not permitted to publish these under our CC-BY 4.0 license, websites are usually intellectual property and are copyrighted.This includes peripheral graphics of the web browser such as icons and button. We ask that you please remove or replace it.

We have now removed the WEPP logo from Fig. 1 to avoid copyright issues for the journal.

6. Please amend your detailed Financial Disclosure statement. This is published with the article. It must therefore be completed in full sentences and contain the exact wording you wish to be published.
1) State the initials, alongside each funding source, of each author to receive each grant. For example: "This work was supported by the National Institutes of Health (####### to AM; ###### to CJ) and the National Science Foundation (###### to AM)." 2) State what role the funders took in the study. If the funders had no role in your study, please state: "The funders had no role in study design, data collection and analysis, decision to publish, or preparation of the manuscript.".

We have added the names of the authors who were awarded the grants and have also clarified the role of the funders in our work.

Reviewers’ Comments

Reviewer - 1

"WEPP: Phylogenetic Placement Achieves Near-Haplotype Resolution in Wastewater-Based Epidemiology" by Pranav Gangwar et al. is an ambitious and timely approach to augment current possibilities of genomic WBE. I think that this contribution does not only serve as a rich inspiration of further research, but is highly relevant to practitioners from the applied public health side. Hence, I would like to strongly support the publication of the manuscript.

We thank the reviewer for the positive and encouraging feedback and appreciate their recognition of the importance and practical relevance of our work.

Having said this, from my point of view, beside minor issues listed below, the manuscript in its current form suffers from two, probably related issues, I would like to see addressed, prior to publication.

We thank the reviewer for the thoughtful comments. We believe we have thoroughly addressed these issues in our revision.

1. I found the use of the terms "lineage", "haplotype", ("genotype",) "allele" confusing. A precise introduction of the terminology in the context of amplicon sequencing of mixed samples would very much improve the readability.

   We thank the reviewer for highlighting this point. To improve clarity, we have now added definitions of the terms lineage, haplotype, and allele as a glossary table (Table 1) in the Introduction.

2. In this context, the term "Potential Haplotypes for unaccounted alleles" is particularly difficult. Related to the latter, I also have to admit that I could not fully follow how the "Read-to-Haplotype Mapping and Potential Haplotypes for unaccounted alleles" as described should produce the promised association with confidence, even across amplicon boundaries. I have observed that in the provided examples analysis the list of Potential Haplotypes is long and no direct measure of (un)certainty is provided. Even if I applaud the ambitious goal to deduce genomic background of unaccounted alleles, since this would be very important applications, I would either like to see a more elaborated evaluation, maybe even in the context of recombination events, or at least to tone down the language a bit.

   We thank the reviewer for this important question and agree that the usage of our terms was confusing. By “Potential Haplotypes for unaccounted alleles”, we were referring to the known haplotypes selected by WEPP that could parsimoniously

explain the unaccounted alleles. In other words, these haplotypes would require the minimal number of additional mutations to optimally align with the reads carrying the unaccounted alleles. To make our term more precise, we have replaced it with “parsimonious haplotype candidates for unaccounted alleles”.

The list of Parsimonious Haplotype Candidates in Fig. 1B is long because there are several identical haplotypes in the MAT to the one it selected. However, as the reviewer rightly suggests, this list can also be long at times due to uncertainty in WEPP’s estimation, for e.g., resulting from inadequate site coverage in the sample. Quantifying this uncertainty with a single metric can be challenging; however, WEPP now provides multiple metrics to help quantify the uncertainty.

(1) WEPP reports a set of ‘Uncertain Haplotypes’ for each selected haplotype (Fig. 1B(i)). To distinguish between the uncertainty arising either from (i) identical haplotypes present in the MAT or (ii) incomplete genome coverage by sequencing reads, WEPP now reports the maximum single-nucleotide distance among all uncertain haplotypes for each selected haplotype candidate (Fig. 1B(i)). This distance is zero when uncertainty is due to identical haplotypes, and non-zero when it results from incomplete coverage. In addition, WEPP reports a genome coverage metric for each selected haplotype (Fig. 1B(i)), indicating the fraction of the haplotype covered by parsimonious read-to-haplotype mapping.

(2) Short sequencing reads contain few informative sites, causing most reads with unaccounted alleles to map parsimoniously to multiple selected haplotypes. We believe that in the absence of additional information, this is a fundamental information limit that constrains confident assignments. To better convey this uncertainty, the dashboard provides a quantitative indicator of uncertainty for these assignments: the *Equally Parsimonious Positions* (EPPs) metric (Fig. 1B(iii)), which reflects the number of selected haplotypes to which a read can be mapped with equal parsimony. Reads containing more informative mutations typically have fewer EPPs (higher confidence), whereas reads with limited information naturally have higher EPPs. These updates are reflected in the manuscript text, Methods, and Fig. 1 caption.

We have elaborated these metrics now in Methods.

To evaluate the “parsimonious haplotype candidates for unaccounted alleles” in the presence of recombinants, we simulated wastewater samples containing a recombinant haplotype alongside haplotypes from its two parental lineages in equal proportions. We considered two recombinants: a Delta–Omicron recombinant (XAY) and an Omicron–Omicron recombinant (XBF), under two conditions: (1) when the recombinant lineage was *known*, i.e., present in the MAT, and (2) when the recombinant was *novel*, i.e., absent from the MAT.

When the recombinant was *known*, WEPP recovered the recombinant and its two parental lineage haplotypes within one single-nucleotide substitution, which typically results in fewer unaccounted alleles (Supplementary Table 12), as expected. In contrast, when the recombinant haplotype was *novel*, WEPP selected the recombinant's two parental lineage haplotypes as candidates, and flagged as unaccounted alleles nearly all informative sites corresponding to the recombinant haplotype, which can serve as evidence for the end user to infer a recombinant. This analysis is now included in Supplementary Results 3.

Minor Issues

1. Fig 2: assuming that the points at x = 0 and y = 0 represent FP and FN, respectively, please elaborate how the different tools perform with respect to correct classification (detection instead of quantification)

   We thank the reviewer for highlighting an important use case of detecting lineages in addition to their abundance estimation that was evaluated in the manuscript. From the data used for Fig. 2, we computed True Positives (TP), False Positives (FP), and False Negatives (FN) for all three tools—Freyja, Pipes et al., and WEPP—across both the simulated and synthetic datasets. These results are now added in the Supplementary Table 11, where we calculated the precision and sensitivity of all the tools. As expected, WEPP demonstrates consistently higher precision and sensitivity than both Freyja and Pipes et al. across both datasets, demonstrating a higher accuracy in lineage detection. We summarize the sensitivity and precision results here for the reviewer's convenience.

**Tool**	**SWAMPy Simulated**		**Illumina-sequenced Synthetic**	
	**Precision**	**Sensitivity**	**Precision**	**Sensitivity**
Freyja	0.25	0.68	0.36	0.64
Pipes et al.	0.22	0.51	0.29	0.95
WEPP	0.58	0.91	0.68	0.95

2. I assume that "All MATs were pre-annotated with PANGO lineage annotations." does only apply to the SARS-CoV-2 tree and not to RSV-A and Mpox trees mentioned before this sentence. If so, please correct and specify the lineage annotation regime used for RSV-A and Mpox.

   We thank the reviewer for pointing out this ambiguity. To clarify, only the SARS-CoV-2 MATs were pre-annotated with both PANGO and Nextclade lineage labels. The Mpox and RSV-A MATs were annotated with only the Nextclade lineages. We have now clarified this distinction in the dataset section of Methods.

3. "For the samples used to mimic realistic cluster emergence scenarios, we first removed the haplotypes found (via ClusterTracker) in the new region from the MAT and simulated 5% of wastewater reads from one of the removed haplotypes, while the remaining 95% were drawn from lineages circulating in the region at that time.". What does "new region" refer to? Please elaborate.

   We thank the reviewer for catching the ambiguity. By “new region”, we refer to the target region into which the haplotypes were introduced during haplotype cluster transmission. For example, in a New York → California transmission scenario, California is the “new region”, whereas in a California → Delaware scenario, Delaware is the “new region”. We have now clarified this in the manuscript to avoid confusion.

4. "For these experiments, we created four base mixtures and simulated each with a novel haplotype at 5%, 10%, or 20% abundance, resulting in 12 samples with 1.6 million reads each." Please specify the nature of simulated haplotypes (number of alleles, distance between alleles).

   We have added a Supplementary Table 13 that describes the number of unique haplotypes, the number of alleles, and the average minimum pairwise distance between haplotypes in each sample, along with WEPP’s lineage abundance estimates for each sample. We have also added the part of the table describing the mixture characteristics below for the reviewer’s convenience. We have also included a summary of these metrics in the Methods section.

Mixture Identifier	Number of unique haplotypes	Number of alleles present in the sample	Avg. minimum distance b/w haplotypes
Mixture_1_5%	91	1018	6.78
Mixture_1_10%	91	1018	6.78
Mixture_1_20%	81	944	6.97
Mixture_2_5%	81	762	4.74
Mixture_2_10%	81	762	4.74
Mixture_2_20%	81	762	4.74
Mixture_3_5%	83	886	4.96
Mixture_3_10%	83	886	4.96
Mixture_3_20%	78	802	4.64

**Mixture_4_5%**	**96**	**957**	**4.95**
**Mixture_4_10%**	**91**	**941**	**5.12**
**Mixture_4_20%**	**81**	**907**	**5.7**

5. Please consider to comment (even if it is just in the discussion) how WEPP would perform in the detection of novel recombinant lineages not present in the MAT used.

   We thank the reviewer for raising this important question. We conducted experiments using two recombinant lineages, a Delta-Omicron recombinant (XAY) and an Omicron-Omicron recombinant (XBF). In each experiment, we simulated a single recombinant haplotype together with haplotypes from its two parental lineages at equal proportions in the sample. We found that for the case when the recombinant lineage was *known*, i.e., present in the MAT, WEPP accurately recovered the proportions of all lineages and their corresponding haplotypes.

   We also evaluated the case when the recombinant was *novel*, i.e., absent from the MAT. This was simulated by removing the recombinant lineage and all of its descendant lineages. In this case, WEPP performs reasonably, selecting the recombinant's two parental lineage haplotypes as candidates, and flagging as unaccounted alleles nearly all informative sites corresponding to the recombinant haplotype, again reinforcing how unaccounted alleles, on top of haplotype selections, can serve as an indicator of novel variants. We have now included this experiment in the Supplementary section on recombinant analysis (Supplementary Results 3) and provided detailed results in Supplementary Table 12, along with incorporating these observations in the Results section.

6. What is the time complexity of the algorithm as a function of tree size and read numbers?

   We thank the reviewer for raising this important question. We performed a runtime analysis of WEPP to characterize its time complexity as a function of both the number of sequencing reads and the number of haplotypes in the MAT (Supplementary Fig. 7). We observed that WEPP's runtime scales sub-linearly with respect to both read count and tree size. This behavior is primarily driven by the read de-duplication optimization, which operates on unique reads rather than total reads and yields substantial performance gains, particularly for short-read sequencing data. In addition, WEPP's region-aware tree search and phylogenetic tree condensation further contribute to the scalability of the algorithm.

7. I did not manage to find the views described in Fig 1B(ii-iv) in the dashboard.

We thank the reviewer for raising this concern. The haplotype and read-level analysis views shown in Fig. 1B(ii–iv) are accessible from the dashboard by clicking on the corresponding nodes in the phylogenetic tree view. This process is also demonstrated in the video tutorial (timestamp: 28 minutes) that we have uploaded to our documentation page.

Reviewer - 2

Gangwar et al. introduce a computational pipeline called WEPP, designed to enhance the resolution and analytical capabilities of wastewater-based epidemiology (WBE). By leveraging phylogenetic placement of sequencing reads onto mutation-annotated trees (MATs), WEPP enables the identification of near-exact viral haplotypes directly from wastewater samples. This approach goes beyond lineage-level detection, allowing for the discovery of novel variants, the inference of multiple introductions, and the early identification of emerging pathogens sometimes weeks before clinical confirmation.

We thank the reviewer for the thoughtful summary.

1. The manuscript states that WEPP "uncovered biological insights missed by other tools" and "detected variants up to five weeks before clinical confirmation," but does not present quantitative performance metrics or confidence intervals. I would suggest to include statistically robust benchmarking with synthetic datasets (such as SWAMPy or Freyja benchmark) and real data tested in parallel with Freyja.

   We thank the reviewer for this thoughtful suggestion and have now added statistical benchmarking for our claims. Specifically, for the claim of early variant detection, we compared WEPP's detected haplotypes of the newly introduced Omicron variant in San Diego during the first three weeks of December 2021 with the earliest corresponding clinical detections (Supplementary Fig. 5) and found that WEPP detected these haplotypes an average of 12.9 days earlier from wastewater than clinical sequencing (with a standard deviation of 17.7 days).

   We also quantified the number of San Diego wastewater datasets containing intra-lineage haplotype clusters and unaccounted alleles that were absent from local clinical sequences (Supplementary Table 14). All 22 datasets (100%) exhibited intra-lineage haplotype clusters, and 20 (90.1%) contained unaccounted alleles not observed clinically. Together, these results imply that WEPP can consistently reveal new biological insights that are missed by existing tools.

2. In addition, although WEPP has been applied to various pathogens, there is no explicit analysis of the impact of coverage and read quality, which are highly variable factors in environmental samples. I recommend including simulated experiments with different levels of coverage and baseline quality (Q-score), evaluating the algorithm's behavior. This analysis would demonstrate the method's robustness in low-quality contexts, common in WBE.

We thank the reviewer for highlighting the important considerations of read quality and coverage for wastewater data analysis. In response, we have added Supplementary Fig. 6, which evaluates how haplotype detection accuracy varies with both read quality (Phred score) and sequencing depth, by simulating reads with SWAMPy. These simulations reveal that, for short-read sequencing, low read quality or sequencing depth can impact WEPP's overall accuracy, but a median Phred score of 38 (default in ART) or above and a coverage of above 25× per haplotype achieves optimal WEPP results. We also note that in most real wastewater datasets, the median Phred score ranged between 37 to 39.

3. The claim that WEPP detects variants up to five weeks before clinical confirmation is highly relevant, but lacks epidemiological context. The inclusion of a time-series table or graph (preferred) comparing WBE detection data with corresponding clinical records should demonstrate the magnitude and consistency of the temporal gain.

   We thank the reviewer for this suggestion. Accordingly, we added Supplementary Fig. 5, which provides a time-series comparison of the earliest wastewater detection of Omicron haplotypes with their earliest clinical collection dates in San Diego during the first three weeks of December 2021, which coincided with the introduction and subsequent spreading of Omicron into the city. We observed that the earliest clinical collection dates lagged behind wastewater detection by an average of 12.9 days (with a standard deviation of 17.7 days), consistent with observations reported in prior studies for lineage detections (Karthikeyan et al., Nature 2022; Sapoval et al., Nature Communications 2023). In real-time settings, the delay in clinical reporting would generally be even longer due to additional processing and reporting delays.

4. How does WEPP account for the impact of recombination events in RNA viruses on phylogenetic placement and downstream haplotype inference? Given that recombination can obscure true evolutionary relationships and lead to misleading branch placements in mutation-annotated trees, it would be important to clarify whether WEPP incorporates any correction, detection, or filtering strategy for recombinant genomes, and how such cases might affect its resolution and interpretation of wastewater-derived sequences.

   We thank the reviewer for raising this important question. We conducted experiments using two recombinant lineages, one Delta-Omicron recombinant (XAY) and one Omicron-Omicron recombinant (XBF). In each experiment, we simulated a single recombinant haplotype together with haplotypes from its two parental lineages at equal proportions in the sample. We found that for the case when the recombinant lineage was *known*, i.e., present in the MAT, WEPP accurately recovered the proportions of all lineages and their corresponding haplotypes. Our results help highlight that even though the placement of the recombinants in the MAT is inaccurate (since they should ideally be placed in a

network rather than a tree), it doesn't affect the ability of WEPP to identify the recombinant haplotypes in the MAT that most closely resemble the ones in the wastewater sample.

We also simulated samples where the recombinant lineage is novel, *i.e.*, when the recombinant lineage and all of its descendants were removed from the MAT. In this case, we observed that WEPP performs reasonably, selecting the recombinant's two parental lineage haplotypes as candidates, and flagging as unaccounted alleles nearly all informative sites corresponding to the recombinant haplotype, again reinforcing how unaccounted alleles, on top of haplotype selections, can serve as an indicator of novel variants.

We have now included these experiments in the Supplementary section on recombinant analysis (Supplementary Results 3) and provided detailed results in Supplementary Table 12, along with incorporating these observations in the Results section.

Reviewer - 3

This manuscript by Gangwar et al. describes a pathogen-agnostic pipeline that can improve wastewater based pathogen surveillance. It is based on phylogenetic placement of sequencing reads onto mutation-annotated trees. Most of the validation in this manuscript was conducted using SARS-CoV-2 sequencing data, with validation extended to RSV-A and Mpox data. The tool enables identification of haplotypes, reporting the abundance of haplotypes and lineages as well as can flag potential novel variants. Data are integrated in an interactive dashboard for visualization. All of these features combined make a powerful package for future application. However, as presented the manuscript is overly long, repetitive at times and a more critical assessment of what the tool can and can't do would be greatly beneficial to the reader.

We appreciate the reviewer's thoughtful analysis and acknowledgment of our work's meaningful contributions. We have addressed the length and repetitiveness by streamlining several sections, especially in the Results, and have expanded our discussion of WEPP's limitations for greater clarity.

1. Summary / last sentence: WWE has been used previously to track lineages and report on variants; please revise that this was previously only restricted to clinical surveillance.

   We thank the reviewer for the thoughtful suggestion. We intended to convey that WEPP enables the detection of emerging variants from wastewater even before their formal lineage designation, an ability previously achievable only through clinical surveillance. Accordingly, we have now modified the statement to include the word 'undesignated' to resolve this ambiguity.

2. Abstract: Statement that WEPP detected findings that previous datasets missed does not seem to be true for all datasets.

   We thank the reviewer for this comment. We agree and have revised these claims to be more accurate, stating that "WEPP uncovered biological insights *sometimes* missed by other tools." We would also like to emphasize that some of WEPP's novel insights, such as identifying: (i) intra-lineage BA.1 haplotype clusters circulating in San Diego (Fig. 4), (ii) multiple independent introductions of B.1.1.529 into San Diego (Supplementary Fig. 3), (iii) mutations indicative of novel variants circulating previously undetected in San Diego (Supplementary Table 6), and (iv) circulating lineages missed by clinical as well as wastewater surveillance methods in Zurich and Geneva (Fig. 5), are epidemiologically significant.

3. Introduction: Starting at paragraph 4 (nearly half of the introduction) the introduction is a summary of the results (even including reference to figures). While a couple of sentences summarizing the approach and results are ok, this is excessive (and the manuscript ends up being repetitive).

   We thank the reviewer for the helpful feedback. We have reduced the description of results in the introduction and streamlined the text to avoid repetition.

4. Results (and also in intro): prediction of subsequent cases is indicated as 2 weeks. This needs context in terms of sampling scale of WW collection as well as likely organism, lineage, immune status of the population. Other reports have indicated a 1 week lag time

   We thank the reviewer for this comment. Several studies cited in our manuscript, including Karthikeyan et al. (Nature 2022) and Sapoval et al. (Nature Communications 2023), have reported a lead time of 1–3 weeks for detecting newly introduced SARS-CoV-2 lineages or variants in a city. We agree with the reviewer that the lead time of wastewater detection is dependent on several factors. Accordingly, we have already mentioned in our manuscript that the early detection could be up to 2 weeks, and have cited the relevant works. Additionally, we respectfully would like to clarify that in this manuscript, we reanalyzed wastewater samples collected in prior studies and demonstrated that, even at the haplotype-level resolution enabled by WEPP, a similar lead time is observed. WEPP has detected haplotypes associated with newly introduced variants an average of 12.9 days earlier than their earliest corresponding clinical collections (with a standard deviation of 17.7 days). This analysis is now presented in the newly added Supplementary Fig. 5 and also described in the Results section.

5. The manuscript refers to high-error ONT sequencing in multiple sections. However, this appears to be an outdated statement given the improvements over the past few years in chemistry and basecalling. Please reword.

We thank the reviewer for correctly pointing this out. The wastewater sequencing datasets used for evaluating WEPP were indeed generated using older ONT flow cells and chemistry (R9.4.1), which have higher error rates than current ONT technologies. We have now clarified this in the Method section, which clearly notes that our analyses are based on legacy, higher-error ONT data rather than the latest and improved lower-error platforms.

6. Repetition: the section on intralineage clusters etc introduces the datasets at the beginning; and then later discusses them; this can be much condensed.

   We thank the reviewer for pointing out the repetition and helping us improve the clarity of the manuscript. In response, we have condensed the text in the Results section describing the intra-lineage cluster analysis to streamline the manuscript.

7. The discussion needs a limitations section. This should also include a discussion on the importance of epidemiologic data and sampling scales (features the tool itself cannot address).

   We thank the reviewer for this helpful suggestion. In response, we have added a dedicated limitations paragraph that discusses key constraints of WEPP, such as its reliance on continuously updated clinical databases and the need for high-quality, sufficiently deep wastewater sequencing to achieve haplotype-level resolution.
